# Differences in the Quantity and Composition of Extracellular Vesicles in the Aqueous Humor of Patients with Retinal Neovascular Diseases

**DOI:** 10.3390/diagnostics11071276

**Published:** 2021-07-15

**Authors:** Yai-Ping Hsiao, Connie Chen, Chee-Ming Lee, Pei-Ying Chen, Wei-Heng Chung, Yu-Ping Wang, Yu-Chien Hung, Chao-Min Cheng, Chihchen Chen, Bei-Han Ko, Min-Yen Hsu

**Affiliations:** 1 Department of Ophthalmology, Chung Shan Medical University Hospital, Taichung 402306, Taiwan; amy1234575@gmail.com (Y.-P.H.); cconnie7@gmail.com (C.C.); cshy1886@csh.org.tw (C.-M.L.); rockinroll355@gmail.com (P.-Y.C.); orien1168@gmail.com (W.-H.C.); bella37245@gmail.com (B.-H.K.); 2 School of Medicine, Chung Shan Medical University, Taichung 402306, Taiwan; 3 Department of Optometry, Chung Shan Medical University, Taichung 402306, Taiwan; 4 Institute of Optometry, Chung Shan Medical University, Taichung 402306, Taiwan; 5 Department of Radiology, Taichung Veterans General Hospital, Taichung 407219, Taiwan; drahcirxp@gmail.com; 6 Department of Ophthalmology, Taichung Veterans General Hospital, Taichung 407219, Taiwan; b92401086@gmail.com; 7 Institute of Biomedical Engineering, National Tsing Hua University, Hsinchu 300044, Taiwan; chaomin@mx.nthu.edu.tw; 8 Institute of Nanoengineering and Microsystem, National Tsing Hua University, Hsinchu 30044, Taiwan; chihchen23@gmail.com; 9 Department of Power Mechanical Engineering, National Tsing Hua University, Hsinchu 300044, Taiwan; 10 Biotechnology Center, National Chung Hsing University, Taichung 402202, Taiwan

**Keywords:** extracellular vesicle, exosome, aqueous humor, nanoparticle tracking analysis, retinal neovascular disease, angiogenesis

## Abstract

Extracellular vesicles (EVs) are secreted by various cells in the body fluid system and have been found to influence vessel formation and inflammatory responses in a variety of diseases. However, which EVs and their subtypes are involved in vascular retinal diseases is still unclear. Therefore, the aim of this study was to explore the particle distribution of EVs in retinal neovascular diseases, including age-related macular degeneration, polypoidal choroidal vasculopathy, and central retinal vein occlusion. The aqueous humor was harvested from 20 patients with different retinal neovascular diseases and six patients with cataracts as the control group. The particle distribution was analyzed using nanoparticle tracking analysis (NTA) and transmitting electron microscopy (TEM). The results revealed that the disease groups had large amounts of EVs and their subtypes compared to the control group. After isolating exosomes, a higher expression of CD81^+^ exosomes was shown in the disease groups using flow cytometry. The exosomes were then further classified into three subtypes of exomeres, small exosomes, and large exosomes, and their amounts were shown to differ depending on the disease type. To the best of our knowledge, this is the first study to elucidate the dynamics of EVs in retinal neovascular diseases using clinical cases. Our findings demonstrated the possible functionality of microvesicles and exosomes, indicating the potential of exosomes in the diagnosis and therapy of retinal neovascular diseases.

## 1. Introduction

Owing to the immune privilege of the eye, ocular diseases are complicated processes that involve inflammation, cell proliferation, transformation and apoptosis, and vascularization of the cornea, iris, retina, optical disc, and choroid. In particular, angiogenesis and vascular leakage are the most common pathological causes of many eye diseases, including age-related macular degeneration (AMD), polypoidal choroidal vasculopathy (PCV), and central vein occlusion (CRVO). 

AMD is an acquired degenerative disease of the central macula that causes significant central visual impairment through a combination of non-neovascular and neovascular derangements. The hallmark of AMD is the presence of yellowish deposits called drusen within the macula beneath the retina and retinal pigment epithelium. They are essentially cellular waste products consisting of lipids and protein deposits. Drusen interfere with the normal retinal blood supply, and along with exosomes can lead to photoreceptor death [1]. In the early stage of AMD, numerous small or intermediate drusen can be seen within the macula, and patients are typically asymptomatic or have minimal visual decline. A rapid deterioration of central vision often presents with an increased loss of photoreceptors and neovascularization over the macular area. Macular neovascularization is often referred to as “wet” AMD, and a lack of neovascularization is referred to as “dry” AMD. People with wet AMD complain of decreased visual acuity, a positive central scotoma, image distortion, and changes in observed object size [2]. Current treatment for AMD is limited because the causal molecular pathways are not understood. Nutritional adjustments and corrective lenses are used as treatment in the early stage, and laser coagulation, photodynamic therapy, anti-vascular endothelial growth factor (anti-VEGF) administration, and visual rehabilitation are used in the advanced stage. Polypoidal choroidal vasculopathy (PCV) is a subtype of AMD with a higher incidence in Asian populations. An elevation in VEGF plays a crucial role in the pathogenesis of PCV. Central retinal vein occlusion (CRVO) occurs when a proximal retinal vein is occluded by a thrombus, and results in elevation of VEGF within the eyeball. Increased VEGF may cause macular edema or vitreous hemorrhage and devastating vision loss without prompt treatment such as intravitreal injections of anti-VEGF antibodies.

However, the response to intravitreal injections of anti-VEGF varies in these neovascular eye diseases, and the incidence rates of early non-responders to intravitreal anti-VEGF injections and tachyphylaxis after serial injections of intravitreal anti-VEGF are high. Due to these therapeutic limitations, further investigations are needed to elucidate the pathogenesis of these neovascular eye diseases.

Extracellular vesicles (EVs) are composed of lipid bilayer-encased extracellular structures, and are mainly classified into exosomes (about 30–150 nm in diameter), microvesicles (about 100–1000 nm in diameter), and apoptotic bodies (about 100–5000 nm in diameter) [3]. Owing to their extensive distribution in a variety of biological fluids, exosomes have attracted the most attention in recent studies. Exosomes are secreted by various cell types, such as cancer cells, endothelial cells, immune cells, stem cells, and retinal pigment epithelial (RPE) cells [4,5], and participate in intercellular signaling and cellular waste management through the release of various bioactive molecules in their lipid bilayer membrane, including lipids, proteins, and genetic materials [6].

The eyes are organs of the visual system with complicated immune regulation processes, and thus various autoimmune diseases such as uveitis, dry syndrome, and optic neuritis can occur after disruption of immune privilege. Previous studies have reported the upregulation of intercellular protein release via exosomes in drusen formation, which is believed to be relevant to the pathophysiology of AMD [1,7]. To protect eye homeostasis, exosomes can cross the blood-retinal barrier and participate in angiogenesis in the retina, which is the major factor involved in the development and progression of sight-threatening eye diseases [8,9] such as autoimmune uveitis, diabetic retinopathy, retinopathy of prematurity, and glaucoma [10]. Additionally, exosomes have been demonstrated to exhibit immunomodulation through the production of a large number of immunoregulatory factors such as transforming growth factor (TGF)-β, interleukin (IL)-4, and IL-10 to modulate the signal transduction of RPE and other cells [11,12], and exert repair activity by promoting ganglion cell survival and glia cell activation [13]. Previous studies have shown the presence of exosomal vesicles between epithelial cells and the stroma after damage to the corneal epithelium [14,15]. In addition, another study reported that intravitreal injections of mesenchymal stem cell exosomes in mice with laser-induced retinal damage reduced macrophage infiltration, retinal ganglion cell apoptosis, and damage to the retina [16].

Taken together, these in vitro and in vivo studies have demonstrated that exosomes are expressed in the eye, and that they may have therapeutic potential in a variety of ocular diseases [17]. However, the role of exosomes in clinical cases with vascular ocular diseases has not been rigorously studied. Moreover, the current exosomal isolation method has been shown to be inaccurate in previous studies, so that the detailed functionality of exosomes remains unclear [18]. Therefore, in this study, the aqueous humor isolated from patients with vascular eye diseases was used to examine the particle pattern and size distribution of EVs and further distinguish their subtypes, including exomeres, exosomes, and microvesicles, to elucidate their pathological roles [19].

## 2. Materials and Methods

### 2.1. Patients

The experiments conducted in the present study followed the principles of the Declaration of Helsinki. All patients signed informed consent before enrollment (IRB number: CS2-19048). To eliminate possible contamination, the aqueous humor of patients was collected by intravitreal injection before phacoemulsification in cataract surgery at Chung Shan Medical University Hospital (Taichung, Taiwan). A total of 20 disease samples were collected from seven patients with AMD, six patients with PCV, and seven patients with CRVO. All patients in the disease group showed disease-specific morphological characteristics, and diagnoses were confirmed after serial examinations. The demographic characteristics of these patients were recorded in Table 1. The aqueous humor collected from the patients with cataracts (n = 6) was used as the control group in the study. None of the patients had undergone any ocular operations within the past 2 months.

### 2.2. Diagnosis of Clinical Lesions

#### 2.2.1. Optical Coherence Tomography (OCT)

Optical coherence tomography (OCT; Spectrails^®^, Heidelberg, Germany) depends on the backscattering of light wavelengths to obtain cross-sectional images of eye tissue, and it provides detailed information with very high resolution. This allows imaging of vessels and retina structures without obscuration by leakage or hemorrhage. In addition, the OCT acquisition time is fast so that repeat scans can be performed, and the high resolution allows for analysis of the flow within a specific axial location of the retina. OCT is considered to be a key instrument in diagnosing vascular eye diseases.

#### 2.2.2. Fluorescein Angiography (FAG)

Fluorescein angiography (FAG) is a procedure lasting for approximately 10 to 20 min in which fluorescein dye is injected into a peripheral vein, and a special camera is used to visualize retinal blood flow and filling conditions by using a confocal scanning laser ophthalmoscope (FAG; Spectrials HRA^+^, Heidelberg, Germany). It is typically used to confirm the diagnosis of vascular eye diseases such as AMD, PCV, CRVO, and branch vein occlusion, and to assess the degree of retinal nonperfusion. FAG can show delayed venous filling, staining of affected retinal veins, and angiographic macular or retinal leakage in retinal vein occlusion diseases of recent and longstanding duration. FAG can be used to identify the presence of collateral vessels, which transfer blood from the retinal circulation to the choroidal circulation. These original vessels are greater in caliber than those of neovascularization and do not show leakage in the late frames of an angiogram. In contrast, neovascular channels such as capillaries present as fine fronds with abundant leakage in the late frames.

#### 2.2.3. Fundoscopic Examination

A fundoscopic examination should be a routine physical examination for every patient with recently diagnosed vascular eye diseases, since the retina is possibly the only part of the vasculature that can be visualized noninvasively. Retinal color fundus photographs were captured by commercial fundus camera (VISCUAM 200, Zeiss, Germany). It can show retinal whitening along with the distribution of occluded vessels, and it is easier to observe clinical lesions such as surface telangiectasia, neovascularization of the optic disc, retinal hemorrhage, and leakage from abnormal appearing vessels. Pupillary dilatation with short-acting cycloplegia before the fundoscopic examination is usually used to dilate the pupil and provide an extensive field of view. It allows imaging of the appearance of the fundus with a fast acquisition time and reduced patient discomfort.

### 2.3. Transmission Electron Microscopy (TEM)

Transmission electron microscopy (TEM) was performed with a JEOL JEM-1400 (Tokyo, Japan) at 120 kV, and using a CCD camera (Erlangshen, Gatan, Pleasanton, CA, USA) to capture images. Samples were washed and dehydrated using graded ethanol solutions of 70%, 80%, 90%, and finally 100%. Thereafter, the dehydrated samples were infiltrated using Spurr-ethanol solutions with 25%, 50%, and 100% resin (30 min/stage). The resin-infiltrated samples were then polymerized at 70 °C, then separated into 70-nm ultrathin sections using a diamond knife and Ultratome (Ultracut S, Leica Reichart, IL, USA).

### 2.4. Nanoparticle Tracking anAlysis (NTA)

The concentration and size of the exosomes were measured using a NanoSight NS300 instrument (NanoSight NS300, Malvern Inc., Worcestershire, UK). An aqueous humor sample was diluted with phosphate-buffered saline (PBS) at 25 °C, and then injected into the sample chamber via a syringe pump. The settings followed the manufacturer’s instructions. Autofocus was adjusted to avoid indistinct particles. Each measurement was repeated six times, and a 60 s video was taken with a frame rate of 30 frames/second; the speed of the syringe pump was set at 40 μL/second, and particle movement was analyzed using NTA software (version 3.2, NanoSight, Salisbury, UK). Each video was analyzed for mean, mode, and concentration. The post-acquisition NTA settings were continuously updated and kept constant between samples.

### 2.5. Exosome Isolation and CD81 Expression

Exosomes were isolated from the aqueous humor samples using an Exoquick exosome isolation kit (System Biosciences, Palo Alto, CA, USA). Exosome characterization was performed using an ExoFlow commercial kit (System Biosciences, Palo Alto, CA, USA) to examine the expressions of common tetraspanins, such as CD81. All procedures were conducted according to the manufacturer’s instructions. Briefly, magnetic beads with anti-CD81 antibodies were prepared and then incubated with isolated exosomes at 4 °C overnight. After removing the excess magnetic beads, the exosomes were stained with fluorescein isothiocyanate (FITC) and examined using a Canto Flow Cytometer (BD Biosciences, San Jose, CA, USA). Positive signals of CD81^+^ exosome expression were expressed as a percentage in the FITC histogram compared with the control group (magnetic beads only). Data analysis was performed using Flowjo 10 software (BD Life Sciences, CA, USA).

### 2.6. Statistical Analysis

Data are expressed as mean ± standard error (SE) in the individual experiments. The Nonparametric Mann–Whitney statistic was used in the amounts of EVs compared to the (control) cataract group. Student’s t-test was used in other results to compare the disease groups with the cataract (control) group. A *p* value < 0.05 was defined as being statistically significant.

## 3. Results and Discussion

### 3.1. Clinical Features of Retinal Diseases

**Figure 1:**
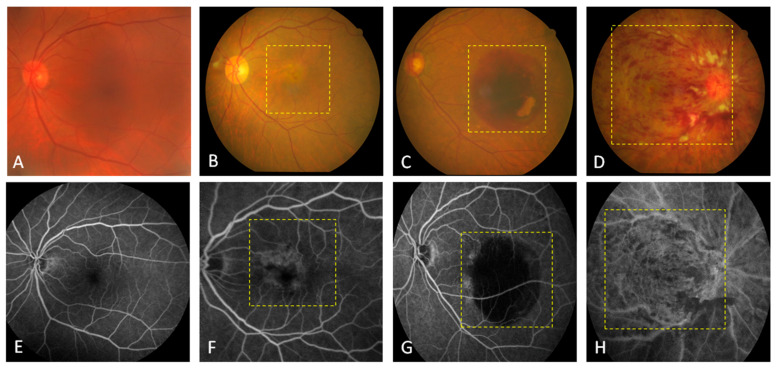
Fundoscopic and fluorescein angiographic examinations of the patients with retinal diseases. Clinical features of the (**A**–**D**) fundus and (**E**–**H**) central retina are presented in the photographs. The fundus photos and fluorescein angiographic (FAG) images showed normal blood flow in a patient with cataracts (**A**,**E**), depigmentation in a patient with wet age-related macular degeneration (AMD; **B**,**F**), a light red patch in a patient with polypoidal choroidal vasculopathy (PCV; **C**,**G**), and diffuse hemorrhage in a patient with central retinal vein occlusion (CRVO; **D**,**H**) in the yellow dashed line boxes.

**Figure 2:**
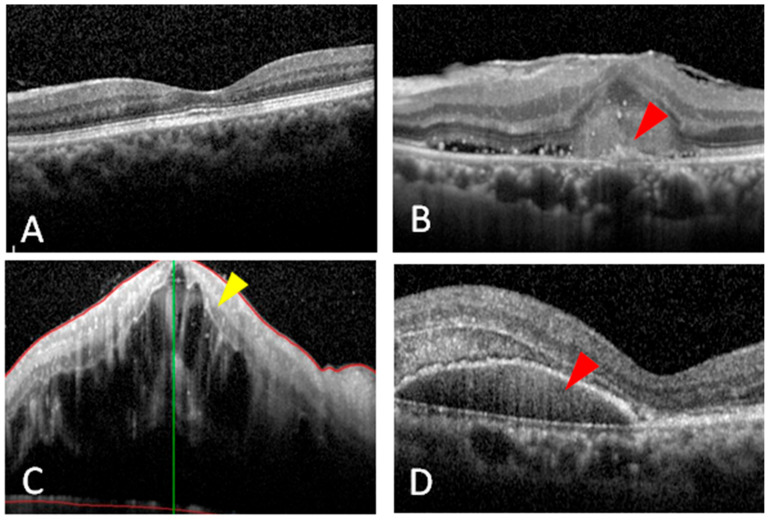
Optical coherence tomography images of the patients with different retinal diseases. (**A**) The image showed a normal macula in the patient with senile cataracts (as the control group). Red arrowheads indicate pigment epithelium detachments and drusen formation in a patient with (**B**) wet age-related macular degeneration and (**D**) polypoidal choroidal vasculopathy. (**C**) The yellow arrowhead shows massive macular edema in a patient with central retinal vein occlusion.

We obtained 20 aqueous humor samples from patients with different retinal diseases (7 with AMD, 6 with PCV, and 7 with CRVO) before undergoing intravitreal injections and six samples from patients before undergoing cataract surgery as controls. Retinal color fundus photographs were used to compare the gross characteristics of these retinal neovascular diseases. The main structures that can be visualized on a fundus photo are the central and peripheral retina, optic disc, and macula. Thus, in the disease groups, the following findings were seen: some depigmentation on the macular area (yellow dashed line box, Figure 1B), a large subretinal hemorrhage (yellow dashed line box, Figure 1C), and diffuse hemorrhage and whitish cotton-wool spots (yellow dashed line box, Figure 1D). All of these characteristics were different from the control group (Figure 1A). FAG is used to evaluate the vasculature of the retina, choroid, and optic disc. All of the patients in the disease groups received FAG, which showed: perimacular leakage of fluorescein dye due to neovascularization of wet AMD (yellow dashed line box, Figure 1F), large halos caused by the blockage of fluorescein due to a PCV-related subretinal hemorrhage (yellow dashed line box, Figure 1G), and diffuse blockage of fluorescein due to CRVO (yellow dashed line box, Figure 1H). There was no leakage in the control group (Figure 1E). OCT is one or even the most important ancillary and detailed tests used to diagnose patients with retinal diseases, and it is the most reliable and reproducible modality to examine pathological changes of the retina. We also acquired OCT images from the patients in the disease and control groups, which showed: drusenoid RPE detachment (red arrowhead, Figure 2B), diffuse intraretinal fluid due to CRVO (yellow arrowhead, Figure 2C), and large subretinal hemorrhage and RPE detachment (red arrowhead, Figure 2D). Intact retinal layers were shown in the control group (Figure 2A).

The studied retinal diseases are well known to be associated with the abnormal development of blood vessels, such as angiogenesis and vessel occlusion. As mentioned above, current treatment strategies are targeted at stopping the overgrowth and/or blockage in vessels by using antibodies against growth factors. However, this is a passive strategy and not effective in every case. Therefore, researchers have tried to elucidate the pathogenesis of retinal diseases to find better strategies for the treatment and diagnosis. EVs are a new frontier in research, and may be a potential treatment target due to their involvement in intercellular communication in retinal diseases [20]. For example, the profiles of inflammatory cytokines in EVs have been shown to be significantly increased in patients with diabetes [21]. Damaged RPE cells have also been reported to release a greater number of EVs with higher expressions of pro-angiogenic factors than non-damaged cells [22]. In addition, circulating EVs from patients with diabetic retinopathy have been shown to express specific cytokines and transcriptional genes in the process of disease and successful treatment [23]. Moreover, human embryonic stem cell EVs have been shown to improve retinal ganglion cell degeneration by rescuing the tauopathy process [24].

Despite these findings, few studies have investigated the association of EVs with retinal diseases, especially CRVO and PCV, and most of these studies have only demonstrated changes in EV proteins in vitro and in vivo. Therefore, in the present study, we used clinical cases with the onset period or without the treatment for 3 months to investigate the amount of EVs and the particle distribution of MVs and exosomes. We harvested the aqueous humor, which is an important intraocular fluid responsible for the supply of nutrients and plays a vital in the maintenance of the optical properties of the eye [25], instead of the vitreous humor to avoid interference of collagen fibers.

### 3.2. Characterization of EVs

**Figure 3:**
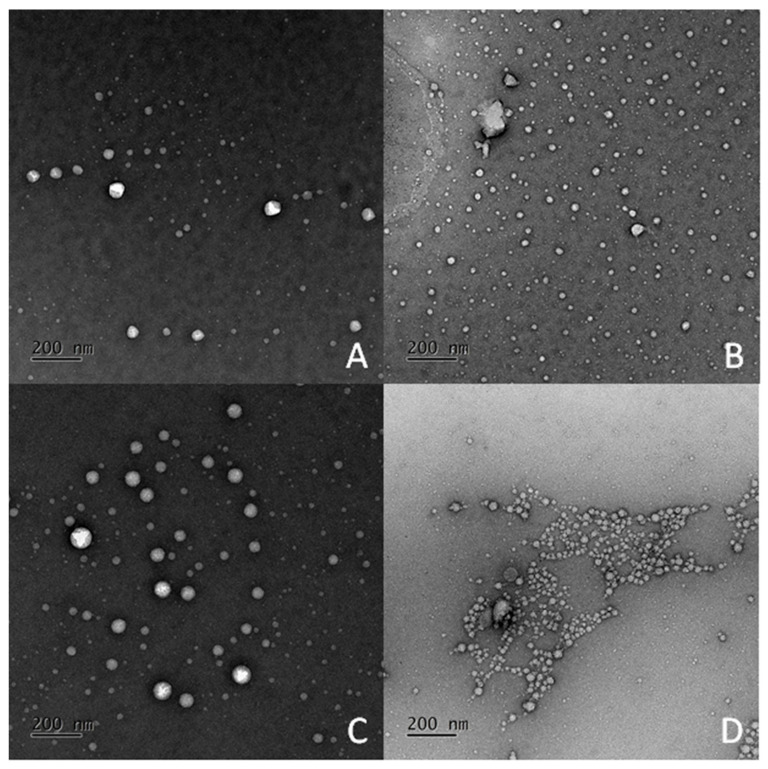
TEM images of extracellular vesicles in patients with ocular diseases. The aqueous humor was harvested from patients with (**A**) cataracts, (**B**) age-related macular degeneration (AMD), (**C**) polypoidal choroidal vasculopathy (PCV), and (**D**) central retinal vein occlusion (CRVO). The images show the representative results of 3 patients per disease. Scale bar indicates 200 nm.

**Figure 4:**
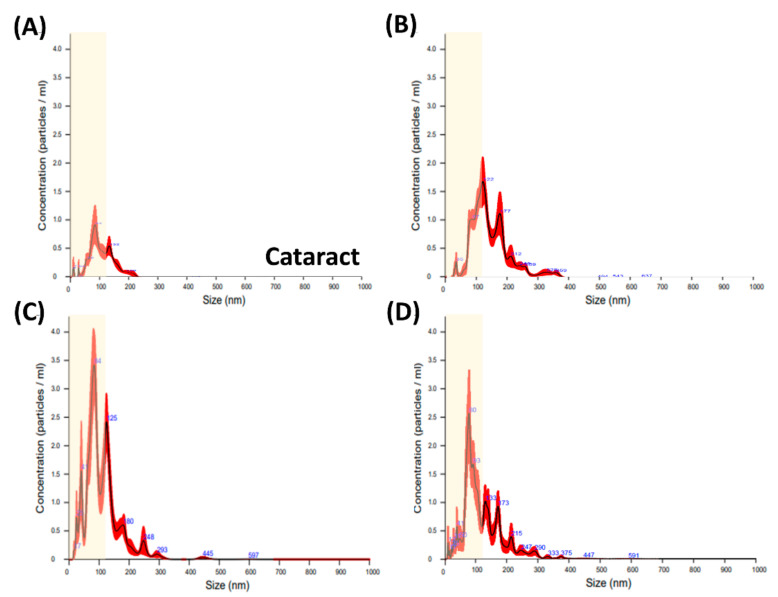
Size distribution of extracellular vesicles from the aqueous humor. The distribution of particle size in the aqueous humor was analyzed using nanoparticle tracking analysis (NTA). The aqueous humor was isolated from the anterior chamber of the patients with eye diseases, including (**A**) cataracts (as the control group), (**B**) age-related macular degeneration (AMD), (**C**) polypoidal choroidal vasculopathy (PCV), and (**D**) central retinal vein occlusion (CRVO). The yellow regions show the range of exosomes (<120 nm) in each disease.

**Figure 5:**
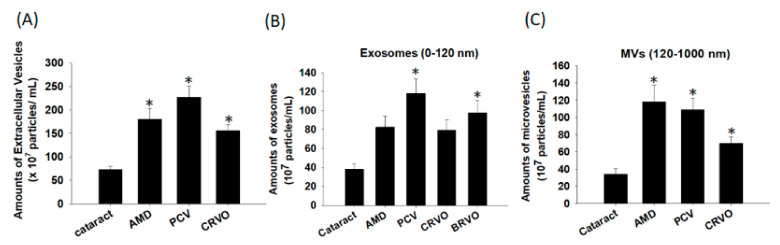
Amounts of microvesicles and exosomes in different retinal diseases. Extracellular vesicles (EVs) were isolated from the aqueous humor of patients with eye diseases, including cataracts, age-related macular degeneration (AMD), polypoidal choroidal vasculopathy (PCV), and central retinal vein occlusion (CRVO), and analyzed using nanoparticle tracking analysis (NTA). (**A**) The results showed the amounts of EVs in the aqueous humors. The EVs were classified into (**B**) exosomes (size of 0–120 nm) and (**C**) other microvesicles (MVs; size of 120–1000 nm). The data are expressed as the mean ± SE of 6–7 samples per group. * *p* < 0.05 compared to the cataract group.

EVs are a general term for secretory vesicles. They are composed of a lipid bilayer membrane and originate from the cellular endosome or plasma membrane and carry diverse cargo including specific proteins, RNA, DNA, and lipids depending on the secreted cell type [3]. Through their role of transporting and exchanging cargo between cells, EVs have been shown to be important mediators of intercellular communication in the maintenance of normal and pathophysiological conditions, such as cancer, neurodegenerative disorders, and infectious diseases [26,27,28]. Although several studies have shown their participation in the development of RPE cells and in murine models associated with eye diseases such as uveitis and AMD [25], the existence of EVs in patients suffering from other eye diseases remains unclear. Therefore, we isolated the aqueous humor from patients with vascular retinal diseases including AMD, PCV, and CRVO, and used the aqueous humor from patients with cataracts as the control group. To examine the presence of EVs, the morphology and particle distribution were examined using TEM. As shown in Figure 3, particles with a round morphology and heterogeneous size distribution were shown in all samples. In addition, greater production and smaller sized particles were observed in the disease groups compared to the control group. Subsequently, NTA, a well-known strategy to analyze particles with a size between 10 nm and 2 µm, at a particle concentration of 10^7^ to 10^9^/mL, was used to quantify the particle distribution of EVs in the aqueous humor samples. The results showed higher amounts of EVs in the disease groups than in the cataract group (Figure 4 and Figure 5A).

### 3.3. Differences in the Particle Size of the EVs

We then analyzed the size distribution of the EVs in the disease groups. The subtypes of EVs have been demonstrated to include several categories, including MVs (50–1500 nm), exosomes (the smallest size; 50–120 nm), apoptotic bodies (50–2000 nm), and blood-derived vesicles (130–500 nm). However, the functionality of these subtypes has yet to be clearly established, and so there is currently no standard classification of these subtypes. Therefore, we used size distribution to distinguish the amounts of exosomes and other MVs as previously reported [29]. In our results, greater production of EVs < 120 nm (defined as exosomes) was noted in the disease groups compared to the cataract group (yellow regions in Figure 4). As shown in Figure 5B,C, the cataract group showed the smallest production of exosomes and MVs, whereas the disease groups showed increased amounts of both types. In particular, the highest expression of exosomes was noted in the PCV group, and the highest expression of MVs was noted in both the PCV and AMD groups. The reason may be due to the biological properties of exosomes and MVs, which are potentially responsible for vessel formation and cell-cell communication. Although MVs and exosomes are structurally similar, there are still some differences in the size and cellular origin. Exosomes are the smallest vesicles released by the fusion of multivesicular bodies containing intraluminal vesicles with the plasma membrane, and possess pleiotropic biological functions on retinal vessels, including modulation of vascular homeostasis, immune activation, and angiogenesis [2,30,31,32]. MVs are heterogeneous vesicles originating from multiple sources, such as selective outward pinching of the plasma membrane to membrane shedding and/or vesicles resulting from cell death [3]. Based on our results, we suggest that MVs in the aqueous humor participate in modulating the inflammatory responses in AMD and PCV, and that exosomes regulate the development of vessels in PCV through the transport of their cargo among endothelial cells, RPE cells, and other cells. This hypothesis is supported by the pathogenesis of these retinal diseases. An increase in oxidative stress-induced inflammation is the main cause of all types of AMD [33], whereas PCV is a variant of wet AMD and associated with both inflammatory responses and also high expressions of VEGF in retina-related cells [34]. The tetraspanin-enriched microdomains of exosomes have consistently been reported to play roles in cellular physics and function distinct from the biological characteristics of lipid rafts, which are expressed on the surface of MVs [35,36]. Therefore, we suggest that exosomes may contribute to the signaling mechanism within intracellular transportation, such as neovascularization, and that MVs assist in the activation of surrounding cells for retinal immunity.

**Figure 6:**
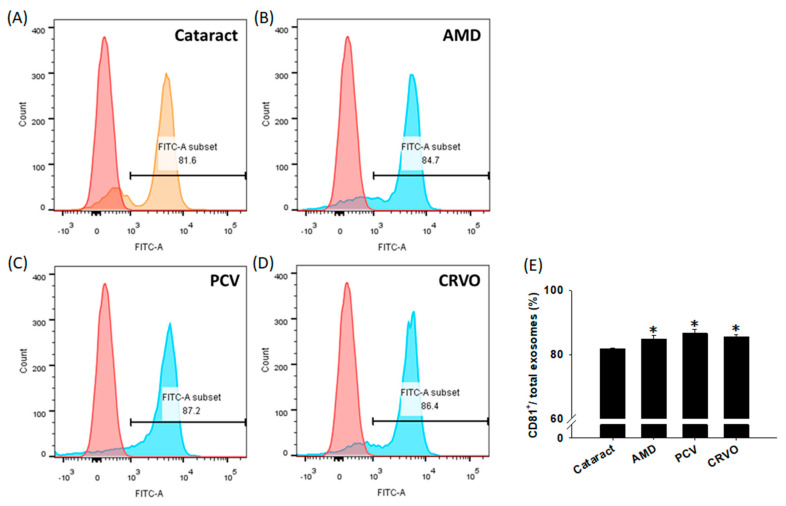
The percentages of CD81^+^ exosomes in ocular diseases. Exosomes were isolated from the aqueous humor (AH) of the patients with cataracts, age-related macular degeneration (AMD), polypoidal choroidal vasculopathy (PCV), and central retinal vein occlusion (CRVO) using a commercial Exoquick kit. The harvested exosomes were stained with CD81^+^ antibodies and analyzed using flow cytometry. (**A**–**D**) Representative histograms show the percentages of CD81^+^ exosomes in ocular diseases. A control group (magnetic beads only and without AH samples) and AH samples from the distinct disease subgroups are shown in red and blue, respectively. (**E**) The quantified results showing the percentages of CD81^+^ exosomes in total exosomes. The data are expressed as the mean ± SE of 3 samples per group. * *p* < 0.05 compared to the cataract group.

Despite these findings, the functionality of exosomes is still controversial due to the overlap in size distribution with other EVs [3]. Previous studies have proposed that exosomes and MVs express a slightly different repertoire of surface receptors or cytoplasmic components. The composition of MVs is closer to the structure of the cell [3], whereas exosomes are enriched with a distinctive subset of cell-derived proteins, nucleic acids, lipids, and glycoconjugates [37]. A study conducted in 1998 was the first to demonstrate that the tetraspanin CD81 is the most highly enriched tetraspanin in exosomes, with a higher expression than CD82, CD37, CD63, and CD9 [38]. As shown in Figure 6, we used CD81 as an exosomal marker to examine the percentage of exosomes in each sample. The cataract group showed the lowest CD81^+^-expressing percentage, while enhanced percentages were noted in the disease groups. The enhanced expression of CD81-bearing exosomes in the disease groups is consistent with the results of exosomal concentrations, indicating the participation of exosomes in the development of vascular eye diseases. Furthermore, these results suggest that future studies may be warranted to investigate the role of CD81 in exosomes.

### 3.4. Size Distribution of Exosomes

**Figure 7:**
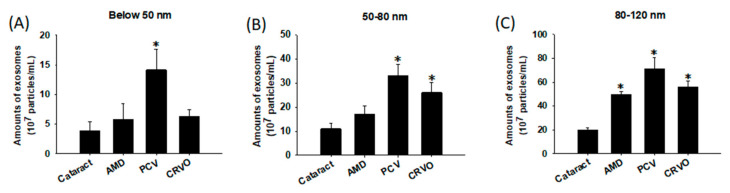
Size distribution of exosomes in different retinal diseases. Extracellular vesicles (EVs) were isolated from the aqueous humor of patients with eye diseases, including cataracts, age-related macular degeneration (AMD), polypoidal choroidal vasculopathy (PCV), and central retinal vein occlusion (CRVO), and analyzed using nanoparticle tracking analysis (NTA). Exosomes were subsequently classified into different size ranges as follows: (**A**) exomeres (below 50 nm), (**B**) small exosomes (Exo-S; 50–80 nm), and (**C**) large exosomes (Exo-L; 80–120 nm), and obtained the amounts of different diseases. The data are expressed as the mean ± SE of 6–7 samples per group. * *p* < 0.05 compared to the cataract group.

Previous studies investigating the biological functions of exosomes have classified them into exomeres (~35 nm) and exosomal subpopulations, including large exosome vesicles (Exo-L; 90–120 nm) and small exosome vesicles (Exo-S; 60–80 nm) [39]. As shown in Figure 7, the cataract group showed the lowest expressions of exomeres and exosomal subpopulations, whereas the PCV group showed the highest expressions among all groups. In addition, increased expressions of both subpopulations were shown in the CRVO group, compared to enhanced production of only the Exo-L subtype in the AMD group. We found that Exo-L may exhibit similar properties with MVs, which have been shown to conduct inflammatory responses in RPE cells. This may be because the population of Exo-L in our study may have contained smaller MVs due to the lucid characterization of MVs and exosomes caused by the overlap in size. Exomeres are a novel population of non-membranous nanoparticles secreted by most types of cells, and their proteins have been reported to be involved in cell metabolism, glycosylation, cytoskeleton, and mitochondrial biogenesis [40]. Combined with our results, this suggests that the pathogenesis of PCV and CRVO is complicated by communication among cells, not merely due to physical damage and inflammation of the retinal cells. However, few studies have investigated exomeres and exosomal subpopulations. To the best of our knowledge, the present study is the first to discover the existence of different sizes of exosomes and MVs in patients with different ocular diseases.

Another unmet need in treating retinal neovascular diseases is to reduce the need for repeat intravitreal injections of anti-VEGF antibodies. Exosomes contain structural RNA, small ribosomal RNA (rRNA), fragmented transfer RNA (tRNA), microRNA, and mRNA [41,42,43,44], and they have been shown to be involved in physiological and pathological processes by transmitting intercellular information and material in cancer patients [45]. Some studies have shown that exosomes can promote angiogenesis through neoplastic processes, protect limbs from ischemic injury, regulate myocardial cell repair and functional recovery in stroke patients [46], and influence VEGF-related signaling pathways [47]. Even though the sample size in the present study is the largest in recent eye disease studies, the volume of aqueous humor was still limited, so we could not examine the expression of VEGF further. Therefore, future investigations with larger clinical samples are needed to examine the detailed regulatory mechanisms of exosomes in vascular retinal diseases in vitro and in vivo.

## 4. Conclusions

Although the isolation of exosomes from the aqueous humor was challenging due to the limited sample per acquisition (~100 microliters per acquisition), our results showed a significant difference in the size distribution of exosomes and MVs between the patients with retinal neovascular diseases and cataracts. These findings suggest that the presence and expressions of MVs (>120 nm) and three different sizes of exosomes, including exomeres (<35 nm), small exosomes (60–80 nm), and large exosomes (90–120 nm), may vary according to the disease type. Further studies are needed to validate the possible diagnostic and therapeutic roles of differently sized exosomes in the pathogenesis of different ocular diseases.

## Figures and Tables

**Table 1:** Demographic characteristics of the group with cataracts (control subjects) and patients with different retinal diseases.

	Cataract	AMD	PCV	CRVO
N	6	7	6	7
Age (years)	72 ± 11	81 ± 3.7	68 ± 6.5	75 ± 19.6
Incipient	6/6	6/7	3/6	5/7
Without treatment for 3 months	-	1/7	3/6	2/7
Smoke	1/6	--	--	--
Hypertension	1/6	3/7	1/6	--
Diabetes	--	2/7	--	2/7
Heart diseases	2/6	1/7	--	1/7

## Data Availability

The data presented in this study are available on request from the corresponding author.
